# Synchronous Multifocal Intraductal Tubulopapillary Neoplasm of the Pancreas Treated with Total Pancreatectomy: A Case Report

**DOI:** 10.70352/scrj.cr.26-0492

**Published:** 2026-09-08

**Authors:** Kohei Suto, Mariko Tsukagoshi, Ayako Yamazaki, Emi Saito, Ryosuke Fukushima, Ryo Muranushi, Kouki Hoshino, Norihiro Ishii, Takamichi Igarashi, Norio Kubo, Kenichiro Araki, Ken Shirabe

**Affiliations:** 1 Division of Hepatobiliary and Pancreatic Surgery, Department of General Surgical Science, Gunma University Graduate School of Medicine, Maebashi, Gunma, Japan; 2 Clinical Department of Pathology, Gunma University Hospital, Maebashi, Gunma, Japan

**Keywords:** intraductal tubulopapillary neoplasm, synchronous multifocal intraductal tubulopapillary neoplasm (ITPN), total pancreatectomy, multicentricity, pancreatic intraductal neoplasm

## Abstract

**INTRODUCTION:**

An intraductal tubulopapillary neoplasm (ITPN) is a rare pancreatic tumor characterized by scant mucin production. Even when associated with an invasive carcinoma component, its prognosis is generally considered more favorable than that of conventional pancreatic ductal adenocarcinoma. However, its pathogenesis and optimal treatment strategy remain unclear. Herein, we report a case of a synchronous multifocal ITPN treated with total pancreatectomy.

**CASE PRESENTATION:**

A man in his 50s was referred to Gunma University Hospital for evaluation of pancreatic duct abnormalities after CT revealed a caliber change in the main pancreatic duct in the pancreatic body and distal pancreatic atrophy. Further evaluation demonstrated lesions associated with main pancreatic duct strictures in the pancreatic body and tail. A diagnosis of resectable pancreatic body and tail cancers was therefore established, and the patient received 2 courses of neoadjuvant chemotherapy (NAC) with gemcitabine plus S-1. Imaging after NAC revealed a new lesion in the pancreatic head. Endoscopic ultrasonography-guided fine-needle aspiration confirmed adenocarcinoma, and the patient was diagnosed with synchronous multifocal pancreatic cancers involving the pancreatic head, body, and tail. Total pancreatectomy was performed. Histopathological examination demonstrated 3 noncontiguous synchronous multifocal ITPNs, each with an independent invasive carcinoma component, in the pancreatic head, body, and tail. The postoperative course was uneventful, and the patient was discharged on POD 15. The patient required no adjuvant therapy and remained recurrence-free at 11 months postoperatively.

**CONCLUSIONS:**

We describe an extremely rare case of a synchronous multifocal ITPN. The noncontiguous distribution observed in this case raises the possibility of multicentric development, although alternative mechanisms cannot be excluded. Further case accumulations and molecular analyses are required to clarify the pathogenesis of this disease and establish optimal treatment strategies.

## Abbreviations

AJCC
: American Joint Committee on Cancer
Bcl-10
: B cell lymphoma/leukemia 10
DUPAN-2
: Duke pancreatic monoclonal antigen type 2
EUS
: endoscopic ultrasonography
FDG
: fluorodeoxyglucose
FNA
: fine-needle aspiration
GS
: gemcitabine plus S-1
HbA1c
: glycated hemoglobin
IPMN
: intraductal papillary mucinous neoplasm
ITPN
: intraductal tubulopapillary neoplasm
NAC
: neoadjuvant chemotherapy
PDAC
: pancreatic ductal adenocarcinoma
SPAN-1
: s-pancreas-1 antigen
SUVmax
: maximum standardized uptake value
TNM
: tumor-node-metastasis

## INTRODUCTION

An ITPN is a rare pancreatic tumor characterized by scant mucin production and tubulopapillary growth within the pancreatic duct, accounting for <1% of exocrine pancreatic neoplasms.^1)^ Even in cases with an invasive component, the prognosis is considered more favorable than that of conventional PDAC.^2)^ ITPN is generally reported as a solitary lesion, and multifocal cases are uncommon.^2)^ Reports of synchronous multifocal ITPN remain extremely scarce. The pathogenesis of multifocal ITPN remains unclear, with proposed mechanisms including multicentric development and intraductal spread. Furthermore, because of its rarity, the optimal surgical strategy for multifocal ITPN has not been established.

Herein, we report a case of synchronous multifocal ITPN involving the pancreatic head, body, and tail treated with total pancreatectomy.

## CASE PRESENTATION

A man in his 50s, with a history of laparoscopic extended left hepatectomy for intrahepatic cholangiocarcinoma approximately 4 years previously, as well as comorbid diabetes mellitus and hypertension, had been undergoing routine follow-up at another hospital. CT performed approximately 1 year before presentation revealed a caliber change of the main pancreatic duct in the pancreatic body and distal pancreatic atrophy. Because these findings progressed, he was referred to Gunma University Hospital for further evaluation and management.

**Fig. 1:**
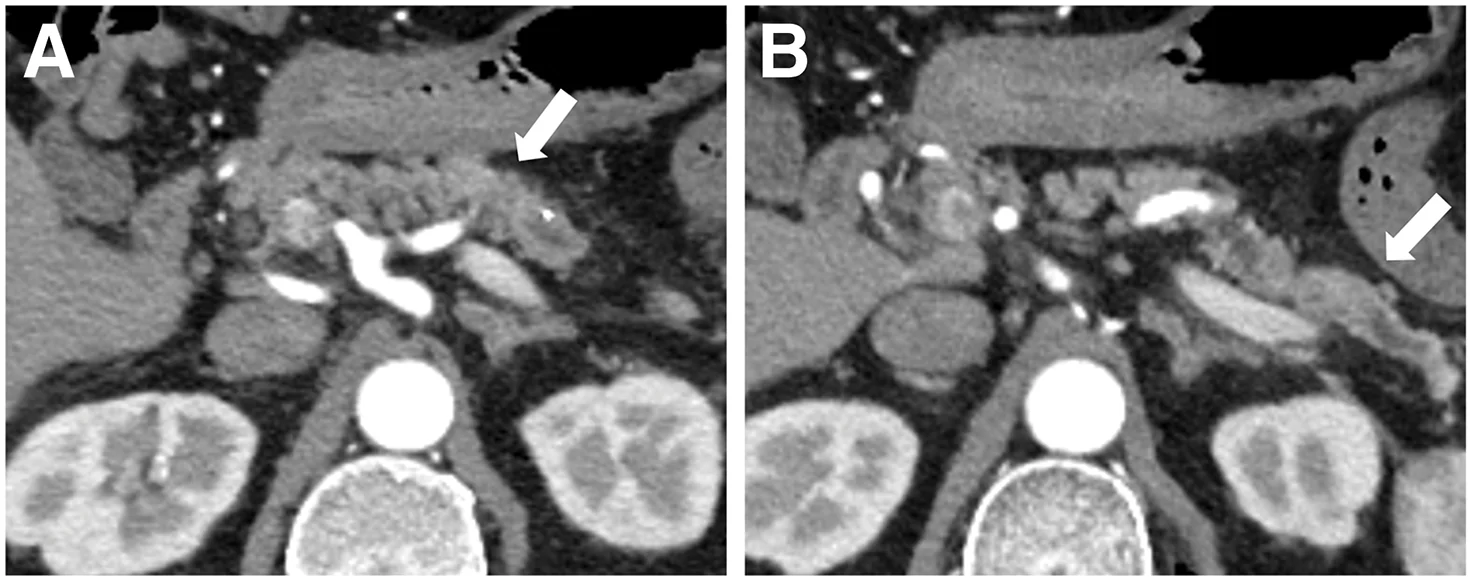
CT imaging findings. **(A)** A main pancreatic duct stricture in the pancreatic body. **(B)** A main pancreatic duct stricture in the pancreatic tail with upstream dilatation of the main pancreatic duct.

**Fig. 2:**
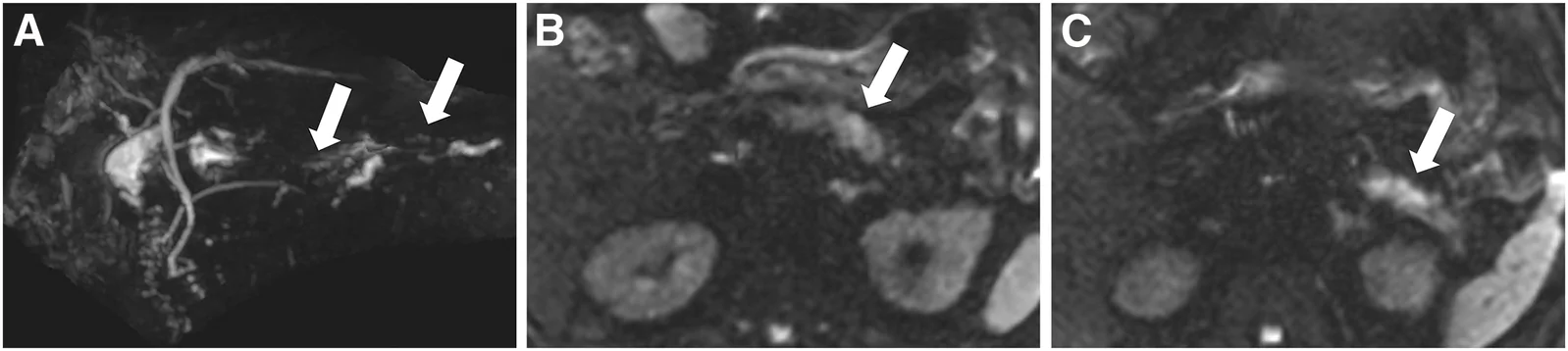
MRI findings. (**A**) Two main pancreatic duct strictures identified on magnetic resonance cholangiopancreatography. (**B**) Diffusion restriction at the site of the main pancreatic duct stricture in the pancreatic body. **(C)** Diffusion restriction at the site of the main pancreatic duct stricture in the pancreatic tail.

Physical examination revealed a flat and soft abdomen with no tenderness or a palpable mass. Laboratory examination indicated an elevated glycated HbA1c level of 7.1%, attributable to his comorbid diabetes mellitus, whereas other hematological and biochemical parameters were within the reference range. Tumor markers, including carcinoembryonic antigens, carbohydrate antigen 19-9, and SPAN-1, were within the reference range; however, DUPAN-2 was elevated at 607 U/mL (reference range, <150 U/mL). Contrast-enhanced CT demonstrated stenosis of the main pancreatic duct in the pancreatic body and tail, along with dilatation of the distal pancreatic duct, although no obvious mass lesion was identified at the site of stenosis (**Fig. 1**). Magnetic resonance cholangiopancreatography revealed dilatation and stenosis of the main pancreatic duct, and diffusion-weighted imaging revealed diffusion restriction corresponding to the stenotic area (**Fig. 2**). EUS revealed a 7 × 8-mm hypoechoic mass in the pancreatic body, and FNA was performed, which demonstrated an atypical epithelium on histopathological examination.

On the basis of these findings, the patient was diagnosed with pancreatic body cancer (cT1cN0M0, cStage IA) and pancreatic tail cancer (cT2N0M0, cStage IB) according to the AJCC TNM classification (8th edition), both of which were considered resectable. Two courses of NAC with GS regimen were administered.

**Fig. 3:**
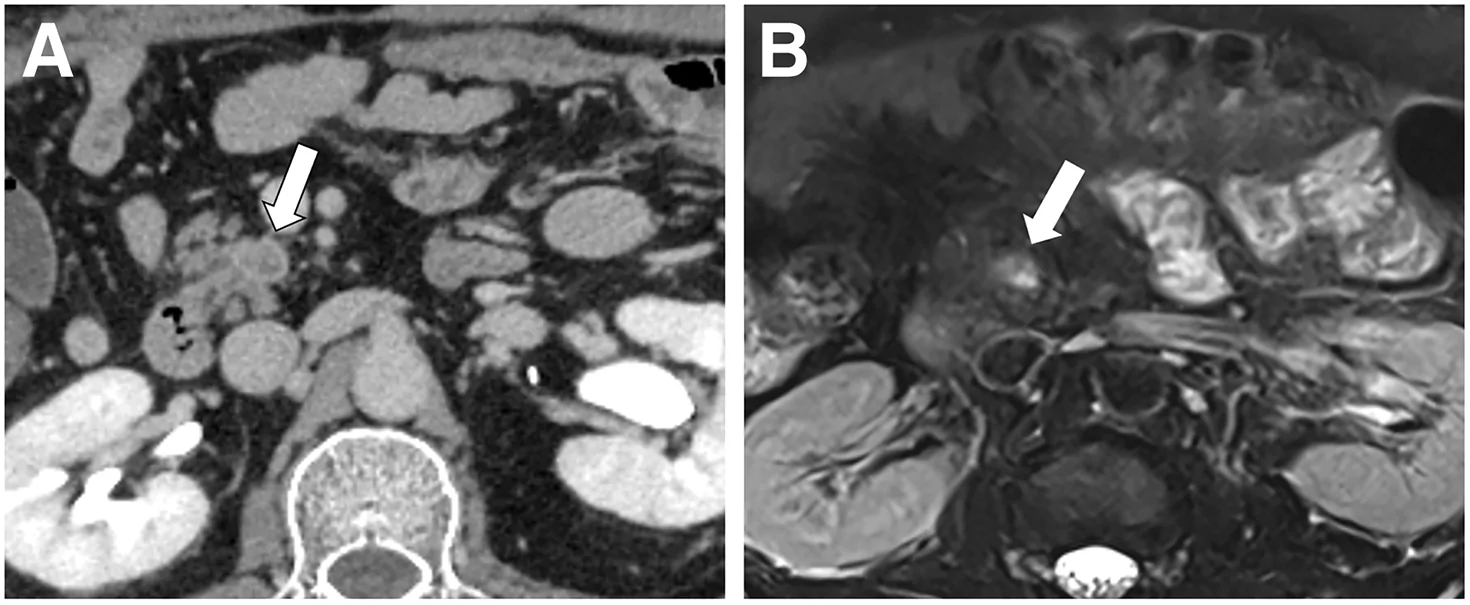
Imaging findings after NAC. (**A**) A newly identified hypoattenuating lesion in the pancreatic head on CT. (**B**) Diffusion restriction at the corresponding site on MRI. NAC, neoadjuvant chemotherapy

**Fig. 4:**
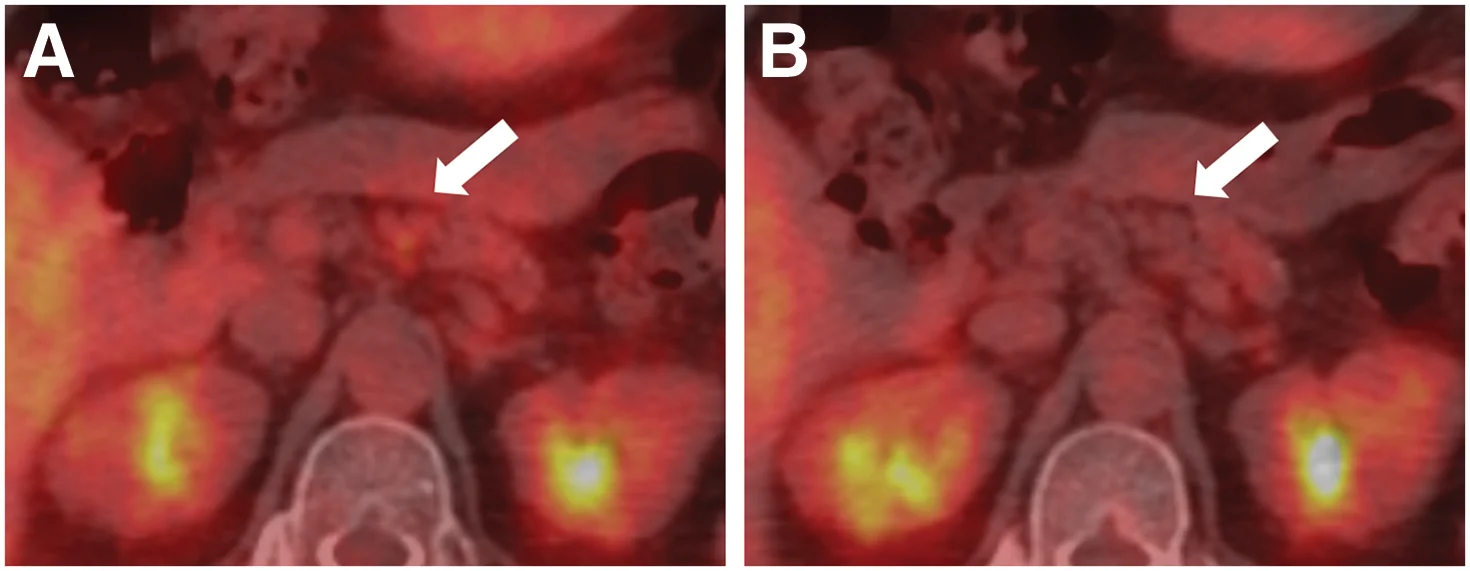
PET-CT findings. (**A**) Mild FDG uptake in the pancreatic body (SUVmax 2.1) before NAC. (**B**) FDG uptake decreased to an SUVmax of 1.5 after NAC. FDG, fluorodeoxyglucose; NAC, neoadjuvant chemotherapy

Following NAC, contrast-enhanced CT revealed a newly detected low-attenuation lesion in the pancreatic head and MRI demonstrated diffusion restriction at the corresponding site (**Fig. 3**). EUS identified hypoechoic masses in the pancreatic head, body, and tail, and FNA of the pancreatic head lesion confirmed adenocarcinoma. On PET-CT, mild FDG uptake was observed in the pancreatic body before NAC (SUVmax 2.1), which decreased to 1.5 after treatment. No significant FDG uptake was observed in the pancreatic head or tail (**Fig. 4**).

The patient was subsequently diagnosed with synchronous multifocal pancreatic cancers: pancreatic head cancer (ycT1cN0M0, ycStage IA), pancreatic body cancer (ycT1cN0M0, ycStage IA), and pancreatic tail cancer (ycT2N0M0, ycStage IB), all considered resectable.

Given the presence of multiple lesions throughout the pancreas, total pancreatectomy with splenectomy was performed to achieve curative resection. The pancreatic tumors exhibited no invasion into the surrounding tissues and were resected without difficulty. However, adhesiolysis around the previous hepatectomy site was required because of dense postoperative adhesions. No apparent metastases to the liver, peritoneum, or regional lymph nodes were observed.

**Fig. 5:**
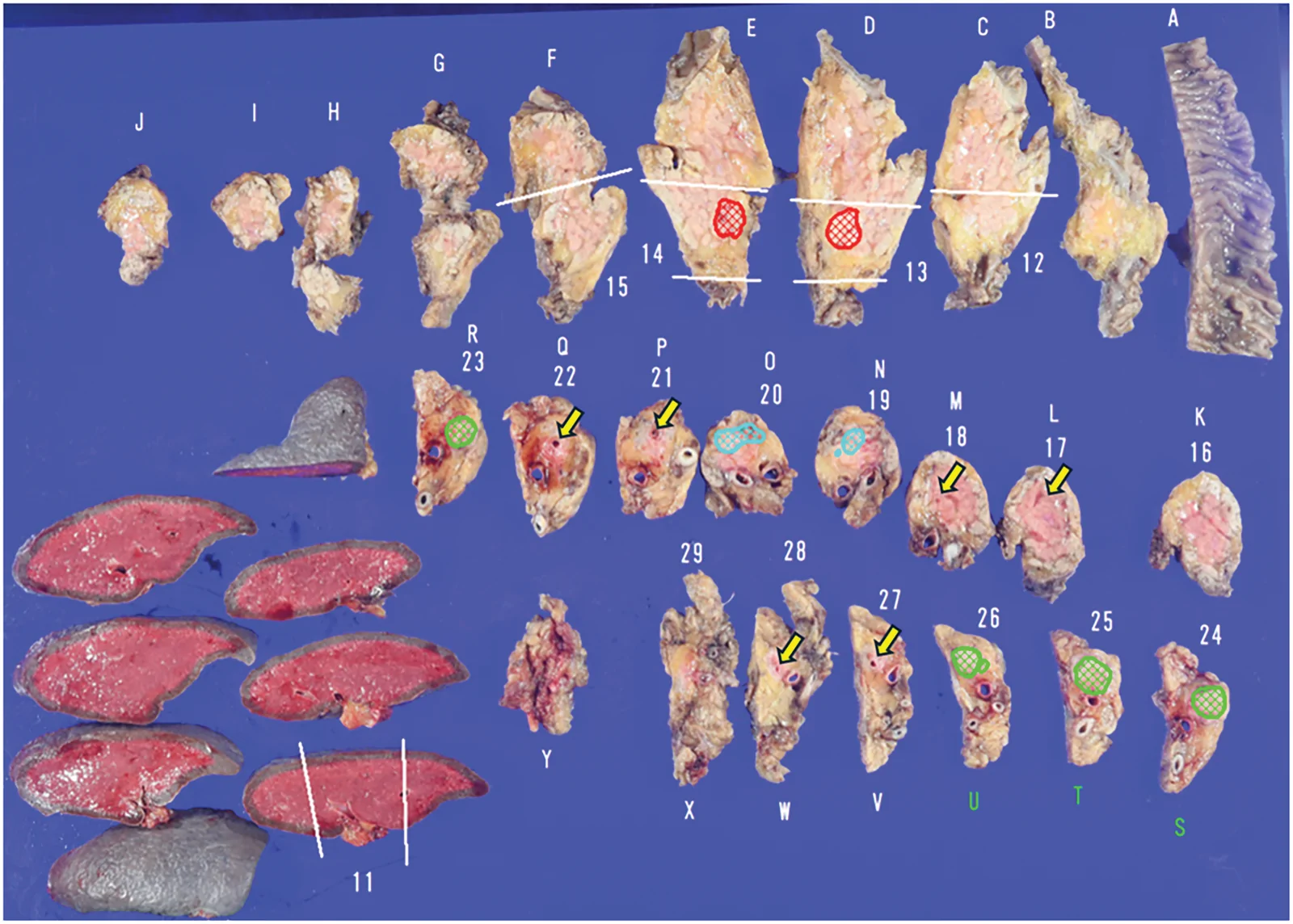
Macroscopic findings of the resected specimen. The tumors measured 1.4 × 1.0 cm (head), 1.3 × 0.6 cm (body), and 2.2 × 1.2 cm (tail). The lesions are outlined in red (head), light blue (body), and green (tail). All lesions proliferated within the main pancreatic duct. No macroscopic continuity was observed between the lesions. Yellow arrows indicate the non-neoplastic main pancreatic duct intervening between the lesions.

**Fig. 6:**
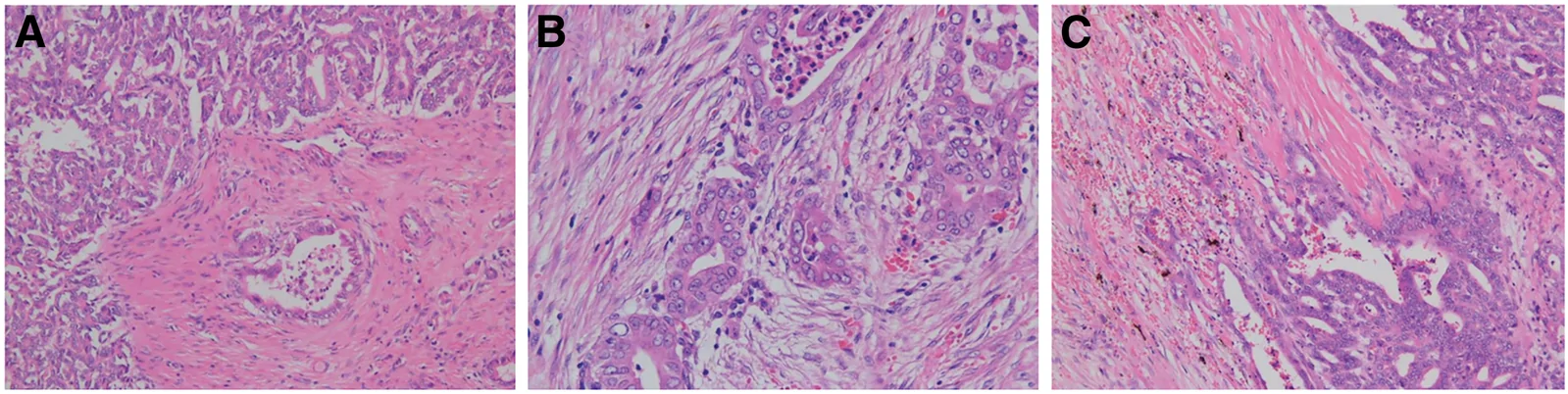
Histopathological findings of the 3 lesions. Tumor cells formed irregular glandular, fused glandular, and papillary structures within dilated pancreatic ducts. (**A**) Lesion in the pancreatic head. (**B**) Lesion in the pancreatic body. (**C**) Lesion in the pancreatic tail. Original magnification: (**A**, **C**) ×100; (**B**) ×200. Hematoxylin–eosin staining.

**Fig. 7:**
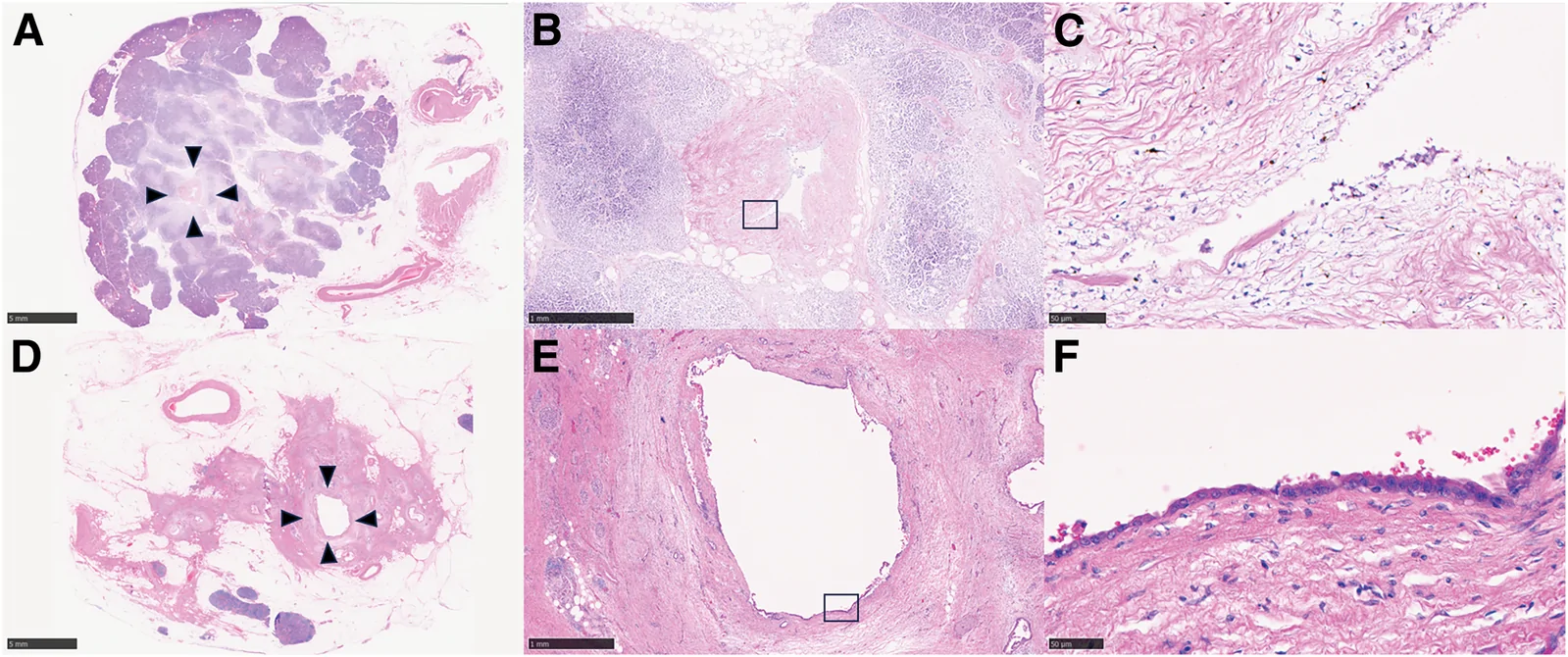
Histopathological findings of the intervening pancreatic duct epithelium. (**A**–**C**) Intervening pancreatic duct epithelium between the pancreatic head and body lesions showing epithelial detachment and degeneration without tumor involvement. (**D**–**F**) Intervening pancreatic duct epithelium between the pancreatic body and tail lesions showing minimal atypia without evidence of neoplastic change. Arrowheads indicate the intervening main pancreatic duct between the lesions in (**A**) and (**D**), demonstrating the absence of tumor continuity. Hematoxylin–eosin staining.

Histopathological examination of the resected specimen revealed solid, grayish-white nodules measuring 1.4 × 1.0 cm in the pancreatic head, 1.3 × 0.6 cm in the pancreatic body, and 2.2 × 1.2 cm in the pancreatic tail, with no macroscopic continuity between the lesions (**Fig. 5**). All lesions demonstrated tumor cells with enlarged round nuclei and an eosinophilic cytoplasm proliferating within dilated pancreatic ducts, forming irregular glandular, fused glandular, and papillary structures, with focal minimal stromal invasion (**Fig. 6A**–**6C**). The invasive components measured 2, 1, and 1.5 mm in the pancreatic head, body, and tail lesions, respectively, and consisted of small nests and trabecular structures. Immunohistochemically, the tumor cells were negative for trypsin and Bcl-10, and no apparent mucin production was observed. These findings were consistent with ITPNs with associated invasive carcinoma components. Microscopically, the lesions were separated by the intervening pancreatic duct epithelium without histological continuity. Between the pancreatic head and body lesions, the ductal epithelium showed detachment and degeneration, but no tumor was identified (**Fig. 7A**–**7C**). Between the body and tail lesions, the ductal epithelium showed minimal atypia without evidence of neoplastic changes (**Fig. 7D**–**7F**).

The final diagnosis was multifocal ITPNs with associated invasive carcinoma involving the pancreatic head, body, and tail. All surgical margins were negative, and no lymph node metastases were identified. The pathological stage was ypT1aN0M0 (ypStage IA) for all 3 lesions. The postoperative course was uneventful, and the patient was discharged on POD 15. No adjuvant therapy was administered. Following total pancreatectomy, endocrine insufficiency was managed with multiple daily insulin injections under the supervision of a diabetologist together with continuous glucose monitoring. Glycemic control was maintained with HbA1c levels in the 8% range. Exocrine insufficiency was managed with pancreatic enzyme replacement therapy. The patient returned to work, remained independent in ADL, and was alive without recurrence 11 months after surgery.

## DISCUSSION

An ITPN is a rare pancreatic tumor characterized by predominant intraductal growth and scant mucin production and is a distinct disease entity separate from other pancreatic neoplasms.^2)^ Unlike IPMN, which exhibits a broad spectrum of dysplasia, ITPN is frequently associated with high-grade dysplasia at the time of diagnosis and may include an invasive component.^1,2)^ However, given its rarity, the pathogenesis and optimal treatment of ITPN remain poorly understood.

Preoperative diagnosis of ITPN is also challenging. Although the lesion may be identified as an intraductal tumor on imaging, its poor mucin production often results in the absence of typical duct-filling features. When the ITPN presents as a hypovascular mass, differentiation from conventional PDAC becomes difficult. In the present case, preoperative imaging demonstrated multifocal main pancreatic duct strictures with upstream duct dilatation but no definite mass lesions at the sites of stenosis. Although these findings may, in retrospect, have suggested an intraductal neoplasm, differentiation from PDAC remained difficult because the imaging findings were not sufficiently characteristic of ITPN. Furthermore, FDG-PET demonstrated only mild uptake in the pancreatic body lesion, and PET findings in ITPN are inconsistent; thus, their utility in distinguishing ITPN from PDAC is considered limited.^2,3)^ In addition, although the pancreatic head lesion was detected only after NAC, it may have been present at the initial presentation but remained radiologically occult. Therefore, its detection after chemotherapy should not be interpreted as evidence of *de novo* tumor development during treatment.

No tumor markers specific to ITPN have been established. In our patient, DUPAN-2 was elevated, which contributed to the suspicion of PDAC; NAC was administered based on this presumption. However, no clear evidence supports the efficacy of chemotherapy for ITPN, necessitating further investigation.

The ITPN is generally considered to spread continuously along the pancreatic duct and is often recognized as a lesion filling the duct on imaging and histopathological examination.^4)^ Therefore, when multiple lesions are identified within the pancreas, it is critical to distinguish whether they represent intraductal extension from a single primary lesion or multicentric development. In the present case, histopathological examination demonstrated an intervening pancreatic duct epithelium without definite neoplastic continuity between the lesions. This finding is difficult to explain by simple intraductal spread alone and raises the possibility of multicentric development. However, alternative mechanisms, including intraductal spread, intrapancreatic metastasis, and intraductal colonization, cannot be excluded.

Other potential mechanisms include intrapancreatic metastasis through lymphatic or hematogenous dissemination. In PDAC, vascular invasion is common, and Fujita et al. reported multifocal intrapancreatic lesions considered to represent intrapancreatic metastases.^5)^ Similarly, Oguro et al. concluded that intrapancreatic metastasis is associated with poorer postoperative outcomes in PDAC.^6)^ In contrast, the ITPN is generally associated with a lower frequency of lymph node metastasis,^1,2)^ suggesting that its biological behavior may differ from that of PDAC.

Another possible explanation is intraductal colonization, in which tumor cells migrate through the pancreatic duct and are implanted at distant sites. This concept has been proposed as a hypothesis to explain spatially separated multifocal lesions in intraductal tumors.^7)^ Ko et al. also discussed the possibility of intraductal colonization in a case of remnant pancreatic recurrence after ITPN resection.^8)^ However, this mechanism has not been firmly established in any intraductal pancreatic neoplasm, and its role in the ITPN, particularly given its poor mucin production, remains unclear.

The present case represents a rare instance of synchronous multifocal ITPN. Most reported cases of multifocal ITPN have been metachronous recurrences, typically involving newly developed lesions in the remnant pancreas after a certain postoperative interval.^2)^ In contrast, synchronous multifocal cases with discontinuous lesions at the time of initial presentation, as observed in this case, are exceedingly rare and provide valuable insights into their pathogenesis. Furthermore, detailed histopathological examination demonstrated an intervening pancreatic duct epithelium without evidence of tumor continuity between the lesions. This finding provides pathological information that has rarely been described in previous reports of synchronous multifocal ITPN and contributes to the discussion of the possible mechanisms underlying multifocal disease.

The multicentric development of ITPN suggests the presence of underlying factors in the pancreatic parenchyma or ductal epithelium that predispose to tumorigenesis. However, unlike IPMN, no clear concept of precursor lesions or field cancerization has been established for ITPN, and the biological basis underlying this phenomenon also remains unclear.

The optimal surgical strategy for multifocal intraductal pancreatic tumors remains controversial. In IPMN, limited pancreatectomy is often considered depending on the degree of dysplasia and the presence of invasive components.^9)^ In contrast, because the ITPN frequently harbors high-grade dysplasia, no standardized surgical approach has been established. Reported cases of the ITPN requiring total pancreatectomy or completion pancreatectomy are summarized in **Table 1**.^8,10–18)^ In previous reports, total pancreatectomy was selected for synchronous multifocal lesions or diffuse intraductal spread involving the entire pancreas to avoid residual disease.^10–13,16,18)^ In addition, several cases required completion pancreatectomy because of newly developed or recurrent lesions in the remnant pancreas after partial pancreatectomy.^8,14,15,17)^

**Table 1:** Reported cases of ITPN requiring total or completion pancreatectomy

No.	Author	Year	Clinical setting	Distribution	Pathological continuity	Invasive component	Procedure	Reason for TP	Outcome/follow-up
1	Kasugai et al.^10)^	2013	ITPN with diffuse intraductal spread	Whole pancreas	Present	No	TP	Extensive intraductal extension	Recurrence-free at 24 months
2	Del Chiaro et al.^11)^	2014	ITPN with diffuse intraductal spread	Whole pancreas	Present	No	TP	Extensive intraductal extension	Follow-up not described
3	Kölby et al.^12)^	2015	Synchronous multifocal ITPN	Whole pancreas	NA	Yes	TP	Partial pancreatectomy deemed infeasible intraoperatively	Recurrence-free at 19 months
4	Fujimoto et al.^13)^	2017	ITPN with diffuse intraductal spread	Whole pancreas	Present	Yes	TP	Partial pancreatectomy deemed infeasible intraoperatively	Recurrence-free at 9 months
5	Saeki et al.^14)^	2018	Remnant pancreatic recurrence after PD for ITPN	Remnant pancreas	NA	Yes	Completion pancreatectomy	Recurrent ITPN in the remnant pancreas	Recurrence-free at 9 months
6	Ko et al.^8)^	2019	Remnant pancreatic recurrence after PD for ITPN	Remnant pancreas	NA	No	Completion pancreatectomy	Recurrent ITPN in the remnant pancreas	Recurrence-free at 23 months
7	Umemura et al.^15)^	2019	Remnant pancreatic recurrence after DP for ITPN	Remnant pancreas	NA	No	Completion pancreatectomy	Recurrent ITPN in the remnant pancreas	Recurrence-free at 4 years
8	Kosmidis et al.^16)^	2020	Synchronous multifocal ITPN	Whole pancreas	NA	Yes	TP	Multifocal ITPN involving the whole pancreas	Liver metastasis at 18 months; alive at 28 months
9	Cohen et al.^17)^	2020	Remnant pancreatic recurrence after PD for ITPN	Remnant pancreas	NA	Yes	Completion pancreatectomy	Recurrent ITPN in the remnant pancreas	Recurrence-free at 8 months
10	Wannasai et al.^18)^	2023	ITPN with diffuse intraductal spread	Whole pancreas	Present	Yes	TP	Extensive intraductal extension	Recurrence-free of ITPN at 3 months
11	Suto et al.	2026	Synchronous multifocal ITPN	Whole pancreas	Absent	Yes	TP	Multifocal ITPN involving the whole pancreas	Recurrence-free at 11 months

NA indicates that pathological continuity could not be evaluated from the published report or was not applicable to recurrent lesions.

DP, distal pancreatectomy; ITPN, intraductal tubulopapillary neoplasm; NA, not applicable; PD, pancreatoduodenectomy; TP, total pancreatectomy

In the present case, discontinuous lesions were distributed throughout the pancreatic head, body, and tail, and partial pancreatectomy was considered likely to result in residual disease. Therefore, total pancreatectomy was selected to achieve curative resection. Although the ITPN is generally associated with a favorable prognosis after resection, Seki et al. reported that recurrence, including liver metastasis and remnant pancreatic recurrence, may occur.^19)^ Therefore, careful long-term surveillance is warranted even after curative resection. At the time of writing, our patient remained alive and recurrence-free at 11 months after surgery; however, careful long-term follow-up is warranted.

This study has several limitations. First, NAC may have induced partial tumor regression, potentially obscuring the original continuity of the lesions. Second, because no molecular analyses were performed, multicentric development could not be definitively distinguished from intraductal spread or intrapancreatic metastasis originating from a single lesion. Further case accumulations and comprehensive genomic analyses are required to elucidate ITPN pathogenesis.

## CONCLUSIONS

We report a rare case of synchronous multifocal ITPN with discontinuous lesions distributed throughout the entire pancreas. Although the distribution of the lesions raises the possibility of multicentric development, other mechanisms cannot be excluded. This case highlights the diagnostic challenge of distinguishing ITPN from conventional PDAC and underscores the importance of appropriate surgical strategy selection in multifocal disease. Further case accumulation and molecular analyses are warranted to elucidate ITPN pathogenesis and identify optimal treatment strategies.

## DECLARATIONS

### Availability of data and materials

All data generated or analyzed during this study are included in the published article.
